# 

**Published:** 1890-12

**Authors:** 


					﻿DECEMBER. 18©O-
OLD SERIES
Vol. XXV
<£*SUs’5£
& 1855 v
MEW SERitf
Vol. VII.—No. I(
Mffl
i hib bT* fa *1*	-	^^axp Rs p* .
H. V. M. MILLER, M.D., LL.D. y
VIRGIL 0. HARDON, M.D.	j
F. W. McRAE, M.D,, M’n’g’ng Ed.
JAMES P.HARR1S0N&.C
SUBSCRIPTIONI 12.00 A YE AIL
				

